# ^1^H-NMR Based Serum Metabolomics Study to Investigate Hepatoprotective Effect of Qin-Jiao on Carbon Tetrachloride-Induced Acute Hepatotoxicity in Rats

**DOI:** 10.1155/2017/6091589

**Published:** 2017-11-01

**Authors:** Zeyun Li, Ying Li, Lingpan Lu, Zhiheng Yang, Wenhua Xue, Xin Tian, Xiaojian Zhang

**Affiliations:** Department of Pharmacy, The First Affiliated Hospital of Zhengzhou University, Zhengzhou 450052, China

## Abstract

Gentiana macrophylla Radix, commonly known as Qin-Jiao (QJ), was recorded alone to treat jaundice in Compendium of Materia Medica and has been frequently prescribed for treatment of liver disease in China. However, the underlying mechanism remains unknown. In the present work, QJ of 1,2 g/kg or silybin of 40 mg/kg (positive control) was orally given to rats for 7 days to verify the protective effect on acute liver damage induced by tetrachloride (CCl_4_). Together with serum biochemistry and histopathological examination, ^1^H-NMR based metabolomics work was carried out to investigate the efficacy. It turned out that QJ of 2 g/kg exerted comparable protective effect with positive control and partially recovered disturbed metabolism by CCl_4_. Multivariate analysis was conducted and metabolites altered significantly among groups were assigned and discussed, including betaine, glucose, lactate, creatine, and LDL/VLDL. Metabolic regulations involved in QJ or silybin treatment were as follows: tricarboxylic acid (TCA) cycle, synthesis of LDL/VLDL, and gluconeogenesis were enhanced, while betaine metabolism, glycolysis, creatine metabolism, synthesis of ketone bodies, amino acids metabolism, and *β*-oxidation of fatty acids were suppressed. For the first time hepatoprotective effect of QJ on acute liver damage was revealed by ^1^H-NMR based metabolomics, prompting understanding of the underlying mechanism.

## 1. Introduction

Liver diseases constitute a global concern and considerable interest has been rising in research of traditional Chinese medicine (TCM) with liver protective efficacy [1, 2]. Gentianae Macrophyllae Radix, dried root of* Gentiana dahurica* Fisch. (Fam. Gentianae) commonly known as Qin-Jiao (QJ), has been an important herb medicine since ancient time in China and was frequently prescribed for treatment of rheumatism, arthralgia, and jaundice [3, 4]. Modern researches have revealed that QJ processed the efficiency of anti-inflammation [5], sedation [6], liver protection [7], and so on. Clinic use of QJ for liver disease has a long history, recorded to be used alone to treat jaundice in Compendium of Materia Medica. However, hepatoprotective efficacy of QJ was not well investigated and the mechanism underlying remains unknown.

Metabolomics, profiling various types of biofluids and reflecting the function and metabolic changes of complete organisms in an holistic view, has attracted a great deal of interest in toxicological [8] or pharmaceutical [9] research. Its holistic view coincides with the integrative thinking of TCM and exhibits great potential in revealing activity, toxicity, and underlying mechanism of TCM [10, 11]. With the advantages of unbiased analysis, simple sample handling, and high reproducibility [12], NMR based metabolomics emerges as one of the most prominent and convenient platforms employed in TCM study [13].

Carbon tetrachloride (CCl_4_) has been extensively used in animal models to explore hepatic injury and to investigate hypoprotective effect of candidates [14]. Many herb medicines or natural products have been proven to exhibit hypoprotective effect on CCl_4_ induced acute liver injury models [15, 16]. In the present work, ^1^H-NMR based metabolomics combined with conventional serum chemistry analysis as well as tissue section examination were carried out to investigate the protective effect of QJ (1,2 g/kg) on acute hepatic damaged rats induced by CCl_4_. Silybin, a well-known natural product with hepatoprotective effect, was given at dosage of 40 mg/kg as positive control. Multivariate data analyses, such as principal components analysis (PCA) and orthogonal partial least-squares discriminant analysis (OPLS-DA), were utilized to reveal pharmacodynamics and to find out related biomarkers responding to liver damage or QJ treatment, which may facilitate the understanding of the pathological changes in acute liver damage and the hepatoprotective mechanisms of QJ.

## 2. Experimental

### 2.1. Chemicals, Reagents, and Herbal Materials Procession

Analytical grade ethanol and carboxymethylcellulose (CMC-Na) were purchased from Sinopharm Chemical Reagent Co., Ltd. (Shanghai, China). D_2_O (99.9% D) was bought from Cambridge Isotoratories, Inc. (Andover, MA). Silybin (purity > 98%) was commercially obtained from Chengdu Biopurify Phytochemicals Ltd. (Chengdu, China). CCl_4_ and coil oil were purchased from Fluka Chimica (Milan, Italy). Kits for serum alanine aminotransferase (ALT) and aspartate aminotransferase (AST) test were purchased from Nanjing Jiancheng Biotech Inc. (Nanjing, China). QJ (*Gentiana dahurica* Fisch.) was purchased from Tong Ren Tang drug store in Zhengzhou (Zhengzhou, China) and identified by chief pharmacist Juan Wang (Department of Pharmacy, The First Affiliated Hospital of Zhengzhou University). A voucher specimen was deposited in the author's laboratory. The roots of QJ (2,000 g) were grinded into powder (<80 mesh) and were extracted with 8 volume of 70% ethanol under reflux (ca. 80°C) for 3 times, each time for 1 hour. After filtering, the filtrates were merged and concentrated to a volume equivalent to 5 g/mL of QJ in 0.3% CMC-Na solution and stored at 4°C.

### 2.2. Animal Experiment and Sample Collection

A total of 35 male Sprague-Dawley (SD) rats (220 ± 20 g, 7 weeks old) were obtained from Hunan Slac Jingda Laboratory Animal Co. Ltd (Changsha, China) and housed at a certified animal experimental laboratory, with a 12 h light/dark cycle and a constant temperature of 25 ± 1°C. Animals were allowed free access to food and water. After acclimatization for 1 week, the rats were randomly assigned to five groups (*n* = 7): a control group (Con), a model group (Mod), a positive control group (SYL), and 2 QJ treated groups (QJ1, QJ2). Con and Mod groups were subjected to oral gavage with 0.3% CMC-Na solution. SYL and QJ treated groups were orally given silybin of 40 mg/kg or QJ of 1,2 g/kg in 0.3% CMC-Na, respectively. Drugs or solvent were administered once a day for 7 consecutive days. 2 h after the last administration, Mod, SYL, and QJ treated groups were intraperitoneally injected with CCl_4_ (2 mg/kg) in corn oil (1 : 1 v/v) to induce acute liver damage, while Con group was let alone. 24 h later, all rats were sacrificed to collect liver tissues and serum samples for histopathological, biochemical assays or metabolomics analysis. Liver tissues were fixed in 10% formalin solution and serum samples were stored at −80°C before analysis.

### 2.3. Histopathology and Serum Biochemistry Assessments

The formalin-fixed liver biopsies were embedded in paraffin wax, sectioned (3-4 *μ*m), and stained with hematoxylin and eosin (H&E) for assessment by the Department of Pathology, The First Affiliated Hospital of Zhengzhou University. Generally, two or five slices were examined for each sample. The serum levels of ALT and AST were tested according to the manufacturer's instructions. Each sample was assayed in duplicate. Statistical analysis was performed using one-way analysis of variance (ANOVA) followed by least-significant difference (LSD) post hoc test (SPSS, Chicago, IL, USA). A probability of *P* < 0.05 was considered to be a statistically significant difference between two groups.

### 2.4. Sample Preparation and ^1^H-NMR Spectroscopy Acquisition

Serum samples were thawed at room temperature, 200 *μ*L of which was mixed with 200 *μ*L D_2_O and 100 *μ*L distilled water. The resulting solution was well mixed and centrifuged at 4°C at 2,000*g* (4,300 rpm) for 5 min to remove any sediment. Then 450 *μ*L of final solution was transferred in a 5-mm NMR tube (Norell, Landisville, NJ, USA) for spectra acquisition.

^1^H-NMR spectra of serum were collected at 298 K on a Bruker 600-MHz AVANCE III NMR spectrometer (Bruker, Germany), equipped with a 5.0-mm BBO probe, operating at 600.13 MHz for ^1^H. The NMR spectrum was recorded using the water-presaturated standard one-dimensional Carr-Purcell-Meiboom-Gill (CPMG) pulse sequence (recycle delay-90°-(г-180°-г)_*n*_-acquisition) to eliminate interference by macromolecules, with a total spin–spin relaxation delay (2*n*г) of 350 ms. A total of 128 free induction decays (FID) were collected into 64 k data points over a spectral width of 10 kHz for each spectrum, with a relaxation delay (D1) of 2 s and power level for presaturation (PLW9) of 33 dB. A line-broadening factor of 0.3 Hz was applied to FID before Fourier transformation. Additional 2-dimensional NMR experiments were performed for the purpose of confirming chemical shift assignments, including homo-nuclear total correlation spectroscopy (2D ^1^H-^1^H TOCSY) and hetero-nuclear single quantum coherence spectroscopy (2D ^1^H-^13^C HSQC) acquired by Bruker's standard pulse sequences mlevphpr and hsqcphpr.

### 2.5. Data Processing and Statistical Analysis

All the NMR spectra were phased and baseline-corrected manually using TOPSPIN 3.0 (Bruker, Germany). The spectra were referenced internally to the chemical shift of creatinine at 3.03 ppm. Each ^1^H-NMR spectrum over the ranged 0.5–9.5 ppm was reduced to 225 regions of equal width (0.04 ppm) and the signal intensity in each region integrated using AMIX (Bruker, Germany). The region of 4.84–5.28 ppm was removed prior to any statistical analysis in order to eliminate any spurious effects of water suppression. Following removal of water regions, data was normalized in AMIX by dividing each integrated segment by the total area of the spectrum to reduce any significant concentration difference. Output data in ASCII data format was imported to Microsoft Excel (Microsoft Office 2007), pareto-scaled, and then imported into SIMCA-P version 13.0.3 (Umetrics, Umeå, Sweden) for multivariate statistical analysis [17].

PCA, a classical unsupervised multivariate pattern recognition method, was employed to examine the intrinsic variation within a group and to assess the clustering behavior among groups. Subsequently, OPLS-DA, a supervised pattern recognition method, was further performed to maximize the variation between each two groups and to determine the variables that contribute to this variation. The quality of models was validated by determining *R*^2^ (goodness of fit parameter) and *Q*^2^ (goodness of prediction parameter) values. The results were visualized in the form of score plots, where each point represents an individual sample (to show the group clusters), or S-plots, where each coordinate represents one ^1^H-NMR spectral region (to identify the variables contributing to the classification). The corresponding variables with variable importance in the projection value (VIP) >1.0 were chosen as major metabolites, whose intensities were compared to indicate the metabolic alteration between groups. Statistical analysis was also performed using ANOVA followed by LSD post hoc test (SPSS, Chicago, IL, USA). A probability of *P* < 0.05 was considered to be a statistically significant difference between two groups.

### 2.6. Identifications of Major Metabolites

According to the ^1^H-NMR spectra of serum samples, major metabolites were assigned by comparison with databases of HMDB (http://www.hmdb.ca; http://www.bml-nmr.org) and the library of the Chenomx software (Vers. 7.6, Chenomx, Edomonton, Canada). Biochemical reactions involving the identified metabolites were found through the Kyoto Encyclopedia of Genes and Genomes (KEGG) and the Human Metabolome Database (HMDB).

## 3. Results and Discussion

### 3.1. Histopathology

**Figure 1:**
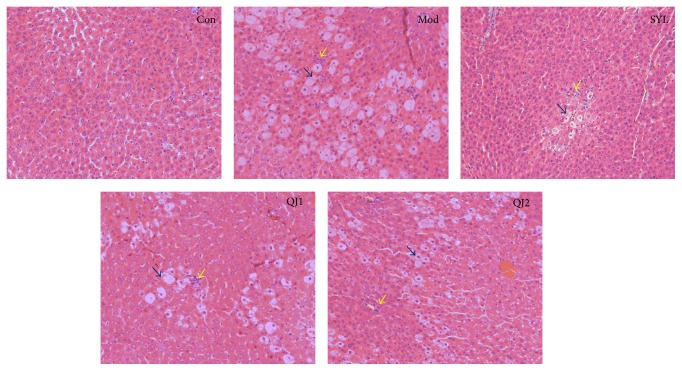
Photomicrographs of the rat liver sections (hematoxylin and eosin staining, ×200).* Note*. Con, control group; Mod, acute liver damage model; SYL, silybin treated group (40 mg/kg); QJ1-2, QJ treated groups (1,2 g/kg); blue arrow marked vacuolation of live cells, and yellow arrow marked inflammatory cell infiltration.

As shown in Figure 1, the histological tissue sections of the Con group showed normal liver histological morphology after H&E staining, while the CCl_4_ induced Mod group exhibit abnormal histopathological changes, with significant vacuolation and degenerative changes in liver, ballooning, and fatty changes of hepatocytes and inflammatory cell infiltration. On the other hand, silybin treated group presents normalization of fatty changes, cellular infiltration, and necrosis, confirming the liver protective effect. The QJ1 (1 g/kg) group showed eliminated vacuolation and inflammatory infiltration, suggesting mild improvement in contrast to Mod group, while the tissue sections of QJ2 group (2 mg/kg) showed significant symptomatic relief compared with Mod group, with vacuolation induced by CCl_4_ remarkably reversed, showing a marked protective effect against CCl_4_-induced liver toxicity. In conclusion, moderate hepatic protection against CCl_4_ was achieved by QJ treatment.

### 3.2. Serum Biochemistry Assessments

Serum aminotransferases, including ALT and AST levels, were used as biochemical indicators of liver damage. As shown in Table 1, serum ALT and AST activities significantly increased in Mod group compared to the Con group (*P* < 0.01), indicating occurrence of liver damage. Not surprisingly, silybin treatment significantly reduced serum ALT and AST by ca. 30% and 60%, in contrast to the Mod group. The reduction was consistent with previous report, explained by the antioxidant capacity of silybin [18]. In parallel, QJ treatment significantly reduced the serum activity level of AST or ALT by 20–60%, which was in line with previous report and can be further supported by similar report of gentiopicroside [19]. Although no significant difference in AST or ALT activities among QJ treated groups was noticed, it seemed that QJ2 group exhibit better reduction of AST or ALT than QJ1 group, inferring better liver protection of 2 g/kg, corresponding to ca. twice the clinical dosage of QJ recorded in Chinese Pharmacopoeia (2015).

### 3.3. ^1^H-NMR Based Metabolomics Analysis

**Figure 2:**
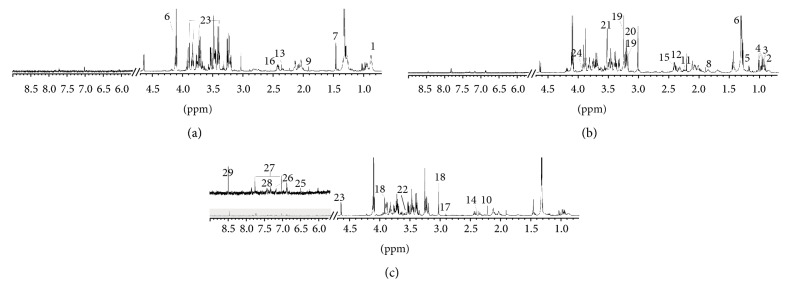
Typical 600 MHz ^1^H-NMR spectra of rat serum from Con (a), Mod (b), and QJ2 (c) groups.* Note*. Con, control group; Mod, acute liver damage model; SYL, silybin treated group (40 mg/kg); QJ2, QJ treated groups (2 g/kg). Metabolites: (1) LDL/VLDL, (2) isoleucine, (3) leucine, (4) valine, (5) 3-hydroxybutyrate, (6) lactate, (7) alanine, (8) lysine, (9) acetate, (10) acetone, (11) acetoacetate, (12) glutamate, (13) pyruvate, (14) succinate, (15) glutamine, (16) citrate, (17) N,N-dimethylglycine, (18) creatine, (19) choline, (20) O-phosphocholine, (21) betaine, (22) glycine, (23) glucose, (24) serine, (25) fumarate, (26) tyrosine, (27) *τ*-methylhistidine, (28) phenylalanine, and (29) format.

Representative serum ^1^H-NMR spectra of Con, Mod, and QJ2 groups were shown in Figure 2, with major metabolites identified. The identified metabolites, chemical shifts, and related metabolic pathways were shown in Table 2. Detailed metabolomics information and differences among groups were revealed by PCA or OPLS-DA model in a more holistic way.

**Figure 3:**
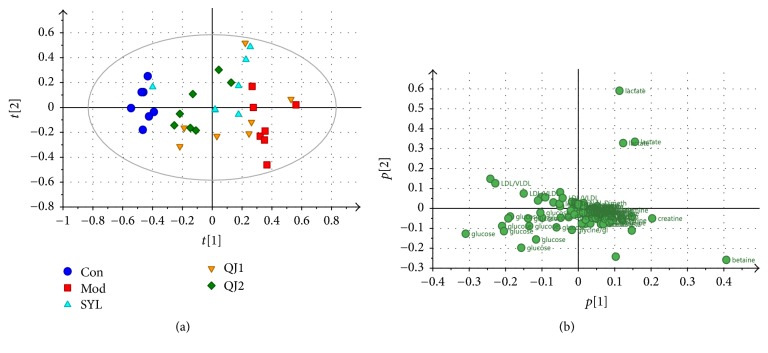
PCA score plot (a), loading plot (b) of serum ^1^H-NMR spectra obtained from Con, Mod, SYL, and QJ1-2 groups.* Note*. Con, control group; Mod, acute liver damage model; SYL, silybin treated group (40 mg/kg); QJ2, QJ treated group (2 g/kg).

PCA score plot of samples from Con, Mod, SYL, QJ1, and QJ2 groups was shown in Figure 3(a), with *R*^2^ 0.846 and *Q*^2^ 0.660. As can be noticed, samples of Mod group clustered far away from those of Con group, indicating successfully established acute liver damage model and confirming histopathology and serum biochemistry result. On the other hand, QJ or SYL treated groups deviated from Mod group and located close to Con group, suggesting that the metabolism disorder of Mod group was partially recovered. In addition, QJ2 group clustered closer to Con group compared with QJ1 group, inferring better protection effect of 2 g/kg. For SYL treatment, the metabolism restoration effect was consistent with previous report [20], and the metabolism change explained histopathology and serum biochemistry result. For QJ treatment, this was the first metabolic revealing of the liver protective effect and may provide further understanding of the protective efficacy. The loading plot (Figure 3(b)) revealed the correlations between groups and variables. Accordingly, glucose and LDL/VLDL were considered to be in high abundance in Con group, while betaine, creatine, and lactate were considered to be characteristics of Mod group.

**Figure 4:**
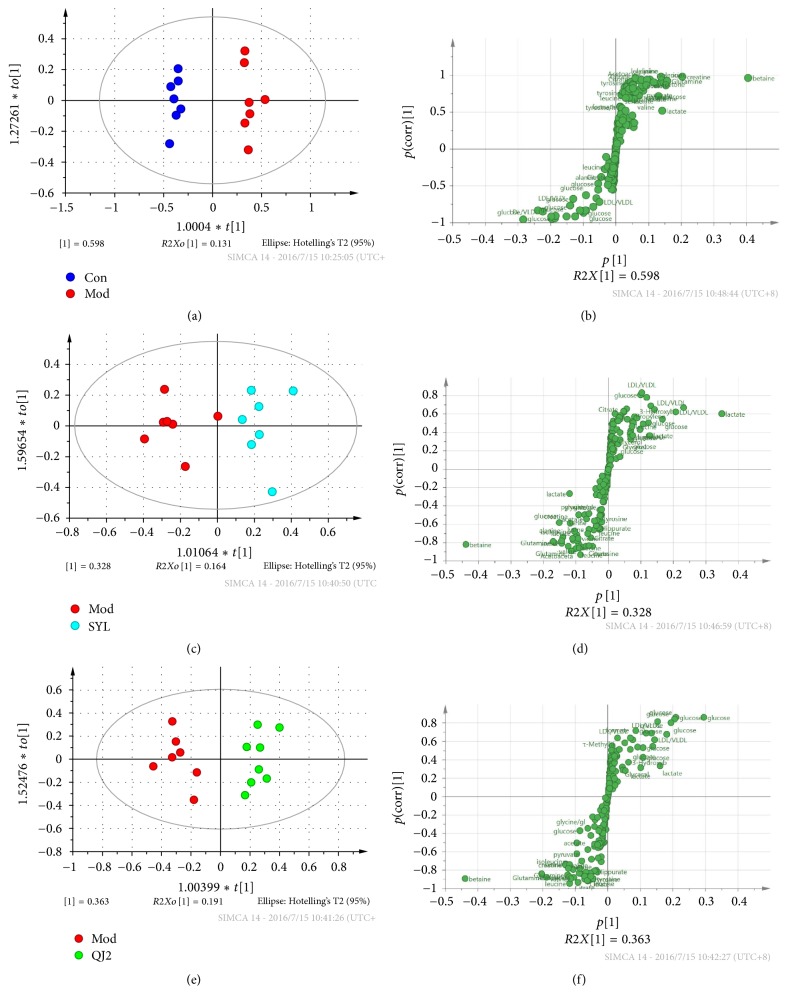
Score plots and S-plots of OPLS-DA model between Con and Mod (a, b), SYL and Mod (c, d), and QJ2 and Mod (e, f) groups.

Subsequently, to explore the metabolic alterations associated with acute liver damage, OPLS-DA model was carried out between Con and Mod groups. The model was well fitted with *R*^2^*Y* 0.979 and *Q*^2^ 0.951 (permutations test result are shown in Figure S1 in Supplementary Material, available online at https://doi.org/10.1155/2017/6091589). The score plot (Figure 4(a)) shows good separation between the two groups, indicating significant metabolism abnormalities. Corresponding S-plot was shown in Figure 4(b), where coordinates in the lower-left quadrant were metabolites significantly increased in Con group compared with Mod group, while those in the upper-right quadrant represent the decreased ones. Meanwhile, VIP values of variables which denote the influence of metabolite on the classification, were calculated by SIMCA-P software. Variables with VIP value >1.0 between two groups were selected and discussed (Table S1). Likewise, OPLS-DA models were carried out between QJ2 and Mod or SYL and Mod groups to reveal the liver protective mechanism of QJ or SYL. The models were well fitted with *R*^2^*Y* and *Q*^2^ value 0.845, 0.572, or 0.918, 0.796 (permutation test is shown in Figure S1). The score plot (Figures 4(c) and 4(e)) shows good separation and confirms the liver protective effect. From the S-plot (Figures 4(d) and 4(f)), metabolites with VIP >1.0 between two groups were selected and listed (Table S1).

**Figure 5:**
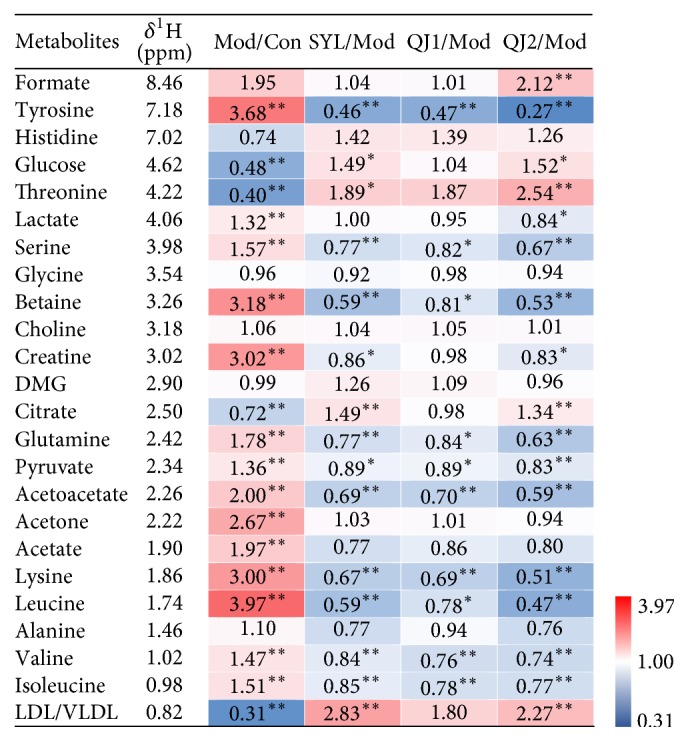
Fold changes of potential biomarkers detected between different groups (*n* = 7). ^*∗*^*P* < 0.05, ^*∗∗*^*P* < 0.01.* Note*. Mod, Con, SYL, and QJ1-2 represent liver damage model, control, silybin (40 mg/kg), and QJ (1,2 g/kg) separately. DMA is short for *N*,*N*-dimethylamine. XXX/YYY means integral of metabolite in XXX group was divided by that of YYY group. The ratio over 1.00 indicated an increase, while ratio less than 1.00 indicated a decrease. Corresponding cell was colored according to the fold change using the color bar labeled at the right side. Statistical analysis was performed by one-way analysis of variance followed by LSD test. ^*∗*^*P* < 0.05; ^*∗∗*^*P* < 0.01.

On the basis of aforementioned OPLS-DA analysis, statistical analysis of potential biomarkers among groups was performed and shown in Figure 5, further detailing alteration trends of metabolites. Alterations and related metabolic pathways were discussed, and it turned out that following metabolic pathways were involved in CCl_4_ induced liver damage and liver protective efficacy: betaine metabolism, synthesis of LDL/VLDL, gluconeogenesis and glycolysis, tricarboxylic acid (TCA) cycle, creatine metabolism, synthesis of ketone bodies, amino acids metabolism, and *β*-oxidation of fatty acids.

Betaine, with the chemical shift of 3.26 ppm and the highest VIP value among each OPLS-DA model, was considered as the most predominant biomarker associated with liver damage or restoration regulations. Betaine comes either from the diet or by the oxidation of choline, with major fate of being phosphorylated or as a donor of methyl-groups. Oxidation of choline to betaine was catalyzed mainly in the mitochondria of liver cells via a series of enzymes, including choline dehydrogenase (CHDH) [21]. CHDL was reported to be related to abnormal mitochondrial function and upregulated in the mitochondrial proteome of rats with fatty liver [22, 23]. The upregulation could be a compensatory response, since betaine is believed to have a hepatoprotective effect [24]. Meanwhile, elevated plasma betaine may promote upregulation of multiple macrophage scavenger receptors or deteriorate liver functions, and thus lead to toxic responses of liver or kidney [25]. In addition, elevated betaine level was also revealed for the toxic responses of processed* Aconitum carmichaelii* Debx. [26]. Consistent with previous reports, in our study, serum betaine level was noticed to increase by 3.18-fold in Mod group, which may be derived from excessive choline released due to CCl_4_ caused membranolysis or elevated levels of related oxidation enzymes. On the other hand, after SYL or QJ treatment, betaine levels were significantly decreased by a half compared with Mod group, which indicated that betaine metabolism disturbed by CCl_4_ inducement was partially restored. The restoration was probably due to the fact that related oxidation enzymes were downregulated.

Another metabolism form of choline was to be phosphorylated, especially as phosphatidylcholine (PtdC), which is necessary for the packaging and export of triglycerides in very low density lipoprotein (VLDL) [27] and for the solubilization of bile salts for secretion [28]. In the case of acute liver damage, betaine may increase at the expense of reduced synthesis of PtdC, and it led to invalid exportation of triglycerides to the blood and accumulation of lipid in the liver. Besides, administration of CCl_4_ causes an increase of triglycerides synthesis in the liver [29]. Enhanced synthesis and unbalanced import and export of triglycerides led to increased lipid and triglycerides levels as characteristic caused by the CCl_4_ administration [30]. As have been verified by histopathological examination, fat accumulation was observed by the formation of foam cells in acute liver damage models. Coincidently, ^1^H-NMR metabolomics analysis has also revealed that serum LDL/VLDL levels were significantly decreased by 70% in Mod group with VIP value of 3.09 in OPLS-DA models of Mod and Con groups. Such alteration was explainable and supported by previous report where VLDL level was found significantly decreased in blood during toxic phase [30]. On the other hand, by SYL or QJ treatment, serum levels of LDL/VLDL were significantly elevated 2-3-fold, revealing that abnormal formation or excretion of triglycerides was restored. The restoration can be attributed to suppressed triglycerides formation or improved excretion of triglycerides from liver. Besides, the increase of LDL/VLDL level may reflect relieve of oxidative stress caused by CCl_4_ administration.

Glucose, with VIP value, was ranked the second primary metabolite altered in acute liver damage models and was decreased by a half in Mod group comparing with Con group. The decrease was consistent with previous report [30], which may be associated with enhanced energy demand and glycolysis, depressed gluconeogenesis. However, by QJ or SYL treatment, serum glucose levels were partially recovered to different degrees, inferring downregulated glycolysis or enhanced gluconeogenesis.

In TCA cycle intermediate, citrate was noticed to decrease in Mod group, indicating suppressed TCA cycle. The suppression was consistent with previous report and the TCA cycle was disturbed by CCl_4_ treatment [31]. On the other hand, suppression of TCA cycle was relieved by QJ or SYL treatment, with citrate level elevated. As end product of glycolysis, lactate was noticed to increase by 30% in Mod group, with VIP value ranked top 4 of all metabolites, reflecting the enhanced glycolysis, which may be explained by increasing energy consumption or reduced plasma oxygen due to inflammatory response, while, after QJ treatment, serum lactate levels were decreased to different degree, especially for QJ2 group with significant improvement, confirming the best liver protective effect of dosage of 2 g/kg. However, SYL treatment did not alter lactate levels compared with Mod group, which may infer that glycolysis was not altered by SYL treatment. This may be difference of regulations by QJ and SYL, and the underlying mechanism remains to be revealed.

Pyruvate, an important intermediate of glycolysis and gluconeogenesis, can enter into TCA cycle or be converted to alanine via alanine aminotransferase (ALT) and to lactate via lactate dehydrogenase (LDH) [32]. In case of acute liver damage, TCA cycle was suppressed, yet glycolysis was enhanced [33]. As previously reported, excess acetyl-CoA accumulated by *β*-oxidization of fatty acid inhibits the utilization of pyruvate, finally leading to elevated levels of pyruvate [32], while after treatment of QJ or SYL serum pyruvate levels were decreased by 20%, suggesting improvement of pyruvate utilization, which may benefit form upregulated TCA cycle or enhanced synthesis of alanine.

Another energy metabolism related metabolite, creatine, was detected to be elevated 3-fold in Mod group and with VIP value ranked 5th metabolites altered between Mod and Con group. As is well known, creatinine is a nonenzymatic breakdown product of creatine and phosphocreatine, and the creatine-phosphocreatine system is crucial for cellular energy transportation [34]. Together with glucose and lactate, alterations of creatine indicated increasing energy demands. More importantly, it is generally known that creatinine is used as a routine detection index for renal dysfunction [35]. Thus, it was indicated that CCl_4_ treatment may further cause kidney function disruption, not just liver disease alone. Interestingly, though not turning back to normal level, QJ or SYL treatment significantly reduced the creatine levels by ca. 20%, inferring mild improvement of energy consumption.

Amino acid metabolisms were also involved in acute liver damage model and protective effect of QJ or SYL treatment. In our study, alanine, valine, leucine, isoleucine, lysine, serine, and glutamine were detected to be altered significantly and with VIP value >1. Comparing Mod group with Con group, serum levels of abovementioned amino acids were elevated, which was in accordance with previous report [36]. Such alterations could be attributed to muscle proteolysis as well as to liver parenchyma necrosis [36]. Besides, alanine, isoleucine, and valine are glucogenic amino acids. The increase in these amino acids indicated that gluconeogenesis was suppressed in case of acute liver damage, partly explaining the decreased glucose level. Leucine, lysine, serine, and tyrosine are ketogenic amino acids, which can be transformed to generate ketone bodies. We have noticed that ketogenic amino acids levels were significantly increased in Mod group, which could indirectly explain the accumulation of ketone bodies. Glutamine can transport ammonia, as it is the mechanism of ammonia transportation and storage [37]. The increase of glutamine level may be associated with an outflow of AST from hepatocellular mitochondrion, proteolysis, or negative nitrogen balance [38]. On the other hand, treatment of QJ or SYL significantly recovered the elevated amino acid levels, with reduction of different degrees. The restoration indicated that abnormal amino acid metabolism was recovered and impaired hepatic regulating function was improved.

Ketone bodies, including acetone and acetoacetate, were produced by the liver from fatty acids and then converted into acetyl-CoA which then enters the TCA cycle. In case of fasting, starvation, or enhanced fatty acids beta-oxidation, synthesis of ketone bodies was upregulated [39]. In current research, serum acetone and acetoacetate levels were detected to increase by 1- to 2-fold in Mod group, which was consistent with previous report where appearance of ketone bodies was considered to be one of the biomarkers for liver injury induced in rats by CCl_4_ exposure [40]. Such increase may be attributed to excessive fatty acid oxidation and serves to eliminate excess amount of acetyl moieties produced. However, after QJ or SYL treatment, serum ketone body levels were detected to decrease, indicating that upregulated synthesis of ketone bodies was suppressed.

Acetate is an end product of fatty acid oxidation, and the doubled serum level of acetate in Mod group further indicate enhanced fatty acids *β*-oxidation [41]. While, by QJ or SYL treatment, serum acetate levels were decreased by ca. 20%, suggesting that acids *β*-oxidation was suppressed, which may also explain the decreased ketone body levels.

**Figure 6:**
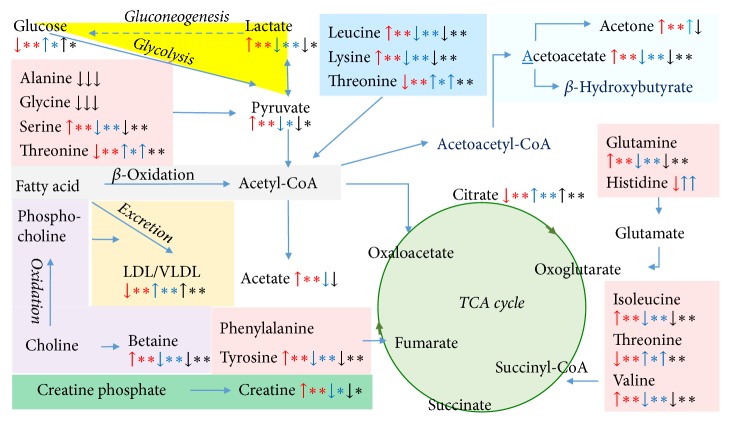
Potential metabolic pathways disturbed in acute liver damage induced by CCl_4_ (red arrow) and restoration regulation by silybin (40 mg/kg, blue arrow) or QJ (2 g/kg, black arrow). ^*∗*^*P* < 0.05, ^*∗∗*^*P* < 0.01.

In summary, QJ treatment partially recovered the abnormal metabolism induced by CCl_4_ administration. QJ of 2 g/kg especially exerts comparable liver protective effect with SYL of 40 mg/kg. According to aforementioned analysis, metabolic pathways regulated by QJ treatment were visualized in Figure 6, with the following metabolic pathways involved: betaine metabolism, excretion of LDL/VLDL, gluconeogenesis and glycolysis, tricarboxylic acid (TCA) cycle, creatine metabolism, synthesis of ketone bodies, amino acids metabolism, and *β*-oxidation of fatty acids.

## 4. Conclusions

In the present work, liver protective effect of QJ was confirmed by serum biochemistry, histopathological examination, and ^1^H-NMR based metabolomics analysis. Metabolites significantly altered among groups were identified and discussed. It turned out that QJ treatment can partially recover abnormal metabolism of liver damage through the following metabolic pathways: TCA cycle, synthesis of LDL/VLDL, and gluconeogenesis were enhanced; while betaine metabolism, glycolysis, creatine metabolism, synthesis of ketone bodies, amino acids metabolism, and *β*-oxidation of fatty acids were suppressed. For the first time ^1^H-NMR based metabolism regulation of QJ on acute liver damage was revealed, which provided further understanding for underlying mechanism and demonstrated ^1^H-NMR based metabolomics as a useful platform for modern research of TCM.

## Supplementary Material

Table S1: VIP value of OPLS-DA models for Con vs Mod, SYL vs Mod, QJ2 vs Mod groups. Figure S1: Permutation test results of established OPLS-DA models. Permutation test was used to check the validity of OPLS models. The intercept is a measure of the overfit. Steep slope indicates well fit. (a) Permutation test for OPLS-DA model of Con and Mod groups; (b) permutation test for OPLS-DA model of SYL and Mod groups; (c) permutation test for OPLS-DA model of QJ2 and Mod groups.

## Figures and Tables

**Table 1:** Serum biochemistry test results (*n* = 7).

	Con	Mod	SYL	QJ1	QJ2
AST	17.83 ± 3.82	515.68 ± 132.64^*∗∗*^	170.11 ± 101.19^*∗∗*^	299.66 ± 97.98^*∗*^	169.44 ± 29.26^*∗∗*^
ALT	19.11 ± 10.56	139.17 ± 58.34^*∗∗*^	108.54 ± 20.33^*∗∗*^	130.79 ± 28.32	121.88 ± 43.21^*∗*^

*Note*. Con, control group; Mod, acute liver damage model; SYL, silybin treated group (40 mg/kg); QJ1-2, QJ treated groups (1,2 g/kg); *∗* means *P* < 0.05, *∗∗* means *P* < 0.01.

**Table 2:** Assignment results of the identified metabolites, chemical shifts, and related metabolic pathways.

Number	Metabolites	Moieties	*δ* ^1^H (ppm) and multiplicity	Related pathway
(1)	LDL/VLDL	CH_2_	0.82(m), 0.86(m), 1.26(m)	Synthesis of LDL/VLDL
(2)	Isoleucine	*γ*CH_3_, *α*CH_3_	0.93(t), 1.00(d)	Gluconeogenesis of glucogenic amino acid/proteolysis
(3)	Leucine	*δ*CH_3_, *δ*CH_3_	0.94(d), 0.96(d)	Ketogenesis of ketogenic amino acids/proteolysis
(4)	Valine	*γ*CH_3_, *γ*CH_3_	1.03(d); 0.97(d)	Gluconeogenesis of glucogenic amino acid/proteolysis
(5)	3-Hydroxybutyrate	*γ*CH_3_	1.19(d)	Synthesis and degradation of ketone bodies
(6)	Lactate	*β*CH_3_, CH	1.33(d), 4.06(q)	Glycolysis and gluconeogenesis
(7)	Alanine	*β*CH_3_	1.46(d)	Gluconeogenesis of glucogenic amino acid/proteolysis
(8)	Lysine	*α*CH_2_, *γ*CH_2_	1.88(m), 1.72(m)	Ketogenesis of ketogenic amino acids
(9)	Acetate	COCH_3_	1.90(d)	*β*-Oxidation of fatty acids
(10)	Acetone	CO(CH_3_)_2_	2.22(s)	Synthesis and degradation of ketone bodies
(11)	Acetoacetate	COCH_3_	2.28(s)	Synthesis and degradation of ketone bodies
(12)	Glutamate	*β*CH_2_	2.34(m)	Glutamate metabolism/nitrogen metabolism
(13)	Pyruvate	COCH_3_	2.37(s)	Glycolysis/gluconeogenesis/TCA cycle
(14)	Succinate	(COCH_2_)_2_	2.40(s)	TCA cycle
(15)	Glutamine	*β*CH_2_	2.40–2.48(m)	Glutamate metabolism/nitrogen metabolism
(16)	Citrate	1/2CH_2_, 1/2CH_2_	2.65–2.70(d); 2.20–2.55(d)	TCA cycle
(17)	N,N-Dimethylglycine	N(CH_3_)_2_	2.90(s)	Glycine, serine and threonine metabolism
(18)	Creatine	N-CH_3_, N-CH_2_-	4.05(s); 3.04(s)	Creatine metabolism
(19)	Choline	N(CH_3_)_3_	3.18–3.19(s)	Choline metabolism
(20)	O-Phosphocholine	N(CH_3_)_3_	3.22(s)	Choline metabolism
(21)	Betaine	N(CH_3_)_3_	3.26(s)	Betaine metabolism
(22)	Glycine	CH_2_	3.54(s),	Glycine, serine, and threonine metabolism
(23)	Glucose	CH	5.20(d), 4.66(d), 3.70–3.90(m), 3.30–3.50(m), 3.84(m)	Glycolysis and gluconeogenesis
(24)	Serine	CH_2_	3.94(d)	Glycine and serine metabolism/ketogenesis of ketogenic amino acids
(25)	Fumarate	CH=CH	6.50(s)	TCA cycle
(26)	Tyrosine	ph-H	7.20(d), 6.86(d)	Tyrosine metabolism/ketogenesis of ketogenic amino acids
(27)	*τ*-Methylhistidine	CH-N-CH=	7.65(s), 7.02(s)	Histidine metabolism
(28)	Phenylalanine	ph-H	7.30(m)	Phenylalanine and tyrosine metabolism
(29)	Formate	HCOO	8.46(s)	Formate metabolism
