# Supplementary material for: 1H-NMR Based Serum Metabolomics Study to Investigate Hepatoprotective Effect of Qin-Jiao on Carbon Tetrachloride-Induced Acute Hepatotoxicity in Rats
Source: Evid Based Complement Alternat Med. 2017 Nov 1;2017:6091589. doi: 10.1155/2017/6091589 (PMC5687146; doi:10.1155/2017/6091589)
Supplement: Supplementary file 1 — Table S1: VIP value of OPLS-DA models for Con vs Mod, SYL vs Mod, QJ2 vs Mod groups. Figure S1: Permutation test results of established OPLS-DA models. Permutation test was used to check the validity of OPLS models. The intercept is a measure of the overfit. Steep slope indicates well fit. (a) Permutation test for OPLS-DA model of Con and Mod groups; (b) permutation test for OPLS-DA model of SYL and Mod groups; (c) permutation test for OPLS-DA model of QJ2 and Mod groups. [file 6091589.f1.zip › Fig.s1.pptx]

## Slide 1
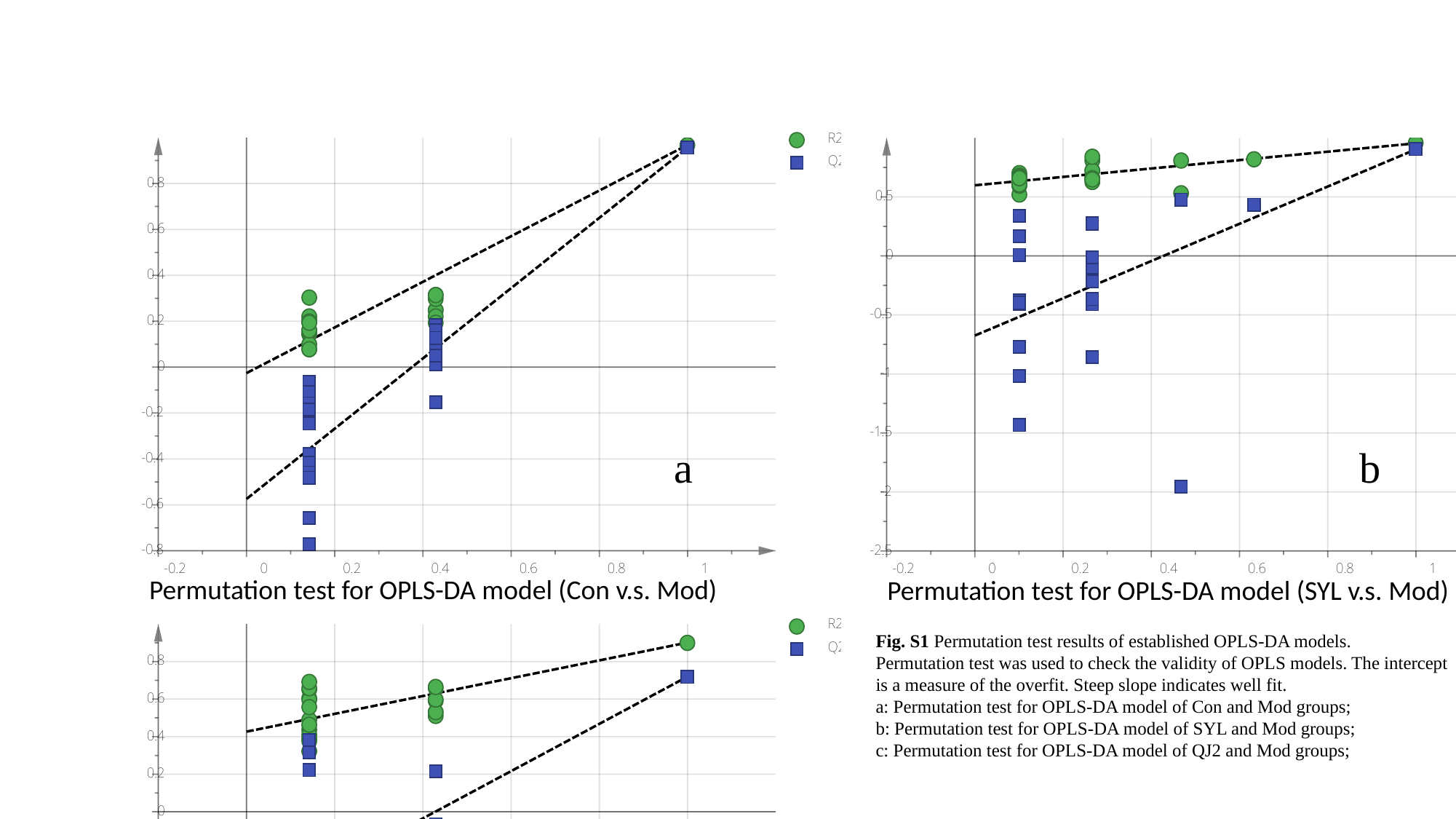

a
b
Permutation test for OPLS-DA model (Con v.s. Mod)
Permutation test for OPLS-DA model (SYL v.s. Mod)
Fig. S1 Permutation test results of established OPLS-DA models.
Permutation test was used to check the validity of OPLS models. The intercept is a measure of the overfit. Steep slope indicates well fit.
a: Permutation test for OPLS-DA model of Con and Mod groups;
b: Permutation test for OPLS-DA model of SYL and Mod groups;
c: Permutation test for OPLS-DA model of QJ2 and Mod groups;
c
Permutation test for OPLS-DA model (QJ2 v.s. Mod)
