# Supplementary material for: 1H-NMR Based Serum Metabolomics Study to Investigate Hepatoprotective Effect of Qin-Jiao on Carbon Tetrachloride-Induced Acute Hepatotoxicity in Rats
Source: Evid Based Complement Alternat Med. 2017 Nov 1;2017:6091589. doi: 10.1155/2017/6091589 (PMC5687146; doi:10.1155/2017/6091589)
Supplement: Supplementary file 1 — Table S1: VIP value of OPLS-DA models for Con vs Mod, SYL vs Mod, QJ2 vs Mod groups. Figure S1: Permutation test results of established OPLS-DA models. Permutation test was used to check the validity of OPLS models. The intercept is a measure of the overfit. Steep slope indicates well fit. (a) Permutation test for OPLS-DA model of Con and Mod groups; (b) permutation test for OPLS-DA model of SYL and Mod groups; (c) permutation test for OPLS-DA model of QJ2 and Mod groups. [file 6091589.f1.zip › Table S1.docx]

**Table S1 VIP value of OPLS-DA models for Con vs Mod, SYL vs Mod, QJ2 vs Mod groups.**

| Metabolites | VIP (Mod/Con) | Metabolites | VIP (SYL/Mod) | Metabolites | VIP (QJ2/Mod) |
| --- | --- | --- | --- | --- | --- |
| Betaine | 5.52 | Betaine | 6.35 | Betaine | 6.10 |
| Glucose | 3.75 | Lactate | 5.15 | Glucose | 3.85 |
| Lactate | 3.29 | LDL/VLDL | 2.79 | Glutamine | 2.65 |
| LDL/VLDL | 3.07 | Glucose | 2.29 | Lactate | 2.49 |
| Creatine | 2.78 | Glutamine | 2.18 | LDL/VLDL | 1.76 |
| Glutamine | 2.11 | Lactate | 2.12 | Isoleucine | 1.70 |
| Alanine | 1.86 | Serine | 1.63 | Lysine | 1.63 |
| Serine | 1.86 | Alanine | 1.54 | Valine | 1.59 |
| Acetone | 1.69 | Creatine | 1.51 | Creatine | 1.55 |
| Isoleucine | 1.58 | Isoleucine | 1.5 | Serine | 1.53 |
| Lysine | 1.43 | Acetoacetate | 1.39 | Acetoacetate | 1.43 |
| Leucine | 1.29 | Lysine | 1.38 | Pyruvate | 1.18 |
| Valine | 1.25 | Leucine | 1.32 | Leucine | 1.17 |
| Acetoacetate | 1.16 | Valine | 1.3 | Alanine | 1.13 |
| Acetate | 1.07 | Glycine | 1.08 | Valine | 1.09 |
